# Functional Polymorphisms in Oxytocin and Dopamine Pathway Genes and the Development of Dispositional Compassion Over Time: The Young Finns Study

**DOI:** 10.3389/fpsyg.2021.576346

**Published:** 2021-04-08

**Authors:** Henrik Dobewall, Aino Saarinen, Leo-Pekka Lyytikäinen, Liisa Keltikangas-Järvinen, Terho Lehtimäki, Mirka Hintsanen

**Affiliations:** ^1^Research Unit of Psychology, University of Oulu, Oulu, Finland; ^2^Department of Psychology and Logopedics, Faculty of Medicine, University of Helsinki, Helsinki, Finland; ^3^Fimlab Laboratories, and Finnish Cardiovascular Research Center - Tampere, Department of Clinical Chemistry, Faculty of Medicine and Health Technology, Tampere University, Tampere, Finland

**Keywords:** oxytocin, prosocial traits, personality development, compassion, dopamine

## Abstract

**Background:** We define compassion as an enduring disposition that centers upon empathetic concern for another person's suffering and the motivation to act to alleviate it. The contribution of specific candidate genes to the development of dispositional compassion for others is currently unknown. We examine candidate genes in the oxytocin and dopamine signaling pathways.

**Methods:** In a 32-year follow-up of the Young Finns Study (*N* = 2,130, 44.0% men), we examined with multiple indicators latent growth curve modeling the molecular genetic underpinnings of dispositional compassion for others across the life span. We selected five single nucleotide polymorphisms (SNPs) whose functions are known in humans: rs2268498 (OXTR), rs3796863 (CD38) (related to lower oxytocin levels), rs1800497 (ANKK1/DRD2), rs4680 (COMT), and rs1611115 (DBH) (related to higher dopamine levels). Compassion was measured with Cloninger's Temperament and Character Inventory on three repeated observations spanning 15 years (1997–2012). Differences between gender were tested.

**Results:** We did not find an effect of the five SNPs in oxytocin and dopamine pathway genes on the initial levels of dispositional compassion for others. Individuals who carry one or two copies of the T-allele of DBH rs1611115, however, tend to increase faster in compassion over time than those homozygotes for the C-allele, b = 0.063 (SE = 0.027; *p* = 0.018). This effect was largely driven by male participants, 0.206 (SE = 0.046; *p* < 0.001), and was not significant in female participants when analyzed separately.

**Conclusions:** Men who are known to have, on average, lower compassion than women seem to reduce this difference over time if they carry the T-allele of DBH rs1611115. The direction of the association indicates that dopamine signaling activity rather than overall dopamine levels might drive the development of compassion.

## Introduction

Compassion can be defined as an enduring disposition that centers upon empathetic concern for another person's suffering and the motivation to act to alleviate it (Lazarus, 1991; Goetz et al., 2010). Experiencing compassion leads to more prosocial behavior, reductions in aggression, and improved intergroup relationships (Eisenberg et al., 2010). Compassion for others as a dispositional personality trait can be understood as the general reactivity of an individual to socio-emotional stimuli (Larsen and Ketelaar, 1991). Garcia et al. (2017) describe compassionate individuals as forgiving, charitable, and benevolent. Individuals characterized as high in compassion usually do not seek revenge and instead try to be constructive in a relationship (Garcia et al., 2017). In interpersonal conflict, compassionate individuals were found to reply with more compromising, integrating, and obliging behavior (Zhang et al., 2014).

From an evolutionary perspective, compassion has emerged from a caring motivation toward the offspring, the kin, and other in-group members, which has an advantage for survival, reproductive fitness, and health (Goetz et al., 2010; Gilbert, 2015, 2020). Compassion for others is consequently essential for forming and maintaining social relationships and building more harmonic societies. What distinguishes compassion from closely related phenotypes, such as empathy and especially empathic concern, is that it is based on the personal desire to help and not the sharing of others' emotions (Lazarus, 1991; Garcia et al., 2017). It is elicited by witnessing undeserved misery of others (Rudolph et al., 2004). Compassion aims at alleviating human suffering and the causes of this suffering, while the adjacent concept of kindness is linked to experiencing joyful emotions (Gilbert et al., 2019). Compassion is, thus, a motivational trait that precedents prosocial behavior (Steffen and Masters, 2005). An emotionally sensitive individual who notices suffering, however, is not inevitably motivated to take action (Poulin, 2017). Compassionate motives require certain competencies to be enacted (Gilbert, 2015). Individuals who understand, for instance, that they share common humanity and hardship with others, and those who are better at tolerating the distress experienced when responding to another person's suffering may also behave more compassionately (Oveis et al., 2010; Klimecki et al., 2014; Lebowitz and Dovidio, 2015; Strauss et al., 2016; Gilbert, 2020). Apart from trait-like compassion that is relatively stable across contexts and time compassion may also refer to a state-like episode or emotion that can change from situation to situation (Goetz et al., 2010). The different aspects of being compassionate are thus context dependent so that an individual may be good at some of them but not necessarily others (Gilbert, 2020). In addition, there is not one way to assess compassion that covers all aspects of it. The current study focuses on dispositional compassion.

### The Genetic Base of Compassion and Closely Related Phenotypes

There is a broad consensus that compassion and compassion-related phenotypes have a significant genetic component (Gillespie et al., 2003; Ando et al., 2004; Knafo and Israel, 2010). A large twin study from Japan, for instance, found that dispositional compassion for others was 34% heritable (Ando et al., 2004). Despite the moderate heritability of dispositional compassion, the contribution of specific candidate genes in its development remains understudied. Only a single small-sample study related candidate genes to dispositional compassion for others (Pełka-Wysiecka et al., 2012), while we can draw on relevant knowledge from studies on empathy and related phenotypes (Gong et al., 2017). Moreover, trait-like compassion might have a different genetic background from compassionate states or the emotion compassion. Focusing on known functional polymorphisms in oxytocin and dopamine signaling pathway genes could offer a potential starting point to shed light on the molecular genetic architecture of dispositional compassion. A polymorphism is referred to as functional if it has been demonstrated that it alters the function of a gene or set of genes (Albert, 2011). The physiological infrastructure that supported the evolution of compassion is not well-understood in humans (Gilbert, 2020); however, it has been suggested that individual differences in compassion are neurologically related to processes of the oxytocinergic and vasopressinergic systems (Carter et al., 2017; Ebert et al., 2018) and dopaminergic reward mechanisms (Klimecki et al., 2013). It is also well-known that the neuropeptide oxytocin has played a vital role in the evolution of compassion's underlying caring motivation (Gilbert, 2020).

The neuropeptides oxytocin and dopamine are known to regulate socio-emotional behavior in general (Ebstein et al., 2012) and compassionate states in particular (Keum and Shin, 2019), and play a putative role in modulating social interaction (Ben-Israel et al., 2015; Pearce et al., 2017). Neuroimaging studies have further found that compassion for other's pain and suffering are related to more activity in oxytocinergic and dopaminergic brain regions (Immordino-Yang et al., 2009; Kim et al., 2009). A recent genome-wide study suggests that allelic variation in the oxytocin and dopamine signaling pathway might indeed be associated with distinct personality profiles that include low vs. high compassion (Zwir et al., 2018).

### Functional Polymorphisms in Oxytocin Pathway Genes

The neurochemical processes in which oxytocin is involved are modulated by a single type of receptor. There are two oxytocin polymorphisms with known functionality. Allelic variation in the oxytocin receptor gene (OXTR) variant rs2268498 was found to have relevance for presumed differences in endogenous oxytocin activity. C-allele carriers showed a 2-fold higher messenger RNA expression rate when compared with individuals with TT genotype (Reuter et al., 2017). Furthermore, the single nucleotide polymorphism (SNP) OXTR rs35062132 was found in functional analyses to cause changes in cellular responses (Ma et al., 2013). Allelic variation in rs2268498 was additionally found to modulate the connectivity of oxytocinergic brain regions (Zimmermann et al., 2018). Melchers et al. (2017) suggested that T-allele carriers, who had better social information processing and memory capacity than individuals with CC genotype, might more easily think and act empathically, and indeed, carrying the TT genotype was associated with decreased threat avoidance (O'Connell et al., 2012) and higher empathic concern, which in turn predicted more prosocial behavior (Christ et al., 2016).

From animal models, it has been known for some time that CD38 (nicotinamide adenine dinucleotide+-glycohydrolase gene) plays an important role in the oxytocin signaling pathway by regulating the secretion of the neuropeptide, which directly influences the plasma oxytocin level (Jin et al., 2007). Feldman et al. (2012) were able to show that individuals with the CC genotype of *CD38* rs379863 have lower plasma oxytocin levels compared with those carrying one or two copies of the A-allele. A-allele carriers, compared with homozygotes of the C-allele, also reported stronger empathic responses leading to more prosocial behavior (Liu et al., 2017).

The soothing and calming qualities of compassion (Pace et al., 2009; Gilbert, 2020), such as not experiencing distress when helping a suffering person, were suggested to be oxytocin mediated (Carter et al., 2017). Oxytocin might thus also be involved in experiencing compassion because of its known role in regulating physiological stress responses (Eisenberg, 2000; Lebowitz and Dovidio, 2015). Of interest here are the parasympathetic autonomic nervous system and its role in conserving energy and calming the body (e.g., decreasing heart rate) and mind. Heightened activation of the vagus nerve has been found to positively correlate with trait-like compassion (Eisenberg et al., 1996) and compassionate states (Stellar et al., 2015). It is therefore not surprising that practicing compassion has been found to decrease an individual's negative affect when dealing with another person's distress (Klimecki et al., 2014) and might improve coping with a stressful situation (Pace et al., 2009; Abelson et al., 2014).

### Functional Polymorphisms in Dopamine Pathway Genes

In the dopamine pathway, three candidate genes with known functional variants have been suggested for compassion-related phenotypes.

In humans, there are five dopamine receptors. The ANKK1 (ankyrin repeat and kinase domain containing 1) polymorphism rs1800497 is located downstream of the dopamine D2 receptor gene (DRD2). Carrying T-allele of this SNP, when compared with homozygotes for the C-allele, results in reduced dopamine receptor-binding density and availability (Pohjalainen et al., 1998) and a reduced relative glucose metabolic rate in dopaminergic brain regions (Noble et al., 1997). The T-allele was found to be associated with an individual's greater need for intimacy and emotional closeness (Gillath et al., 2008), to make the brain's reward centers less reactive (Stice et al., 2008), as well as to modify the effects of stressful life events (Elovainio et al., 2007). ANKK1/DRD2 rs1800497 appears to be related to self-reported empathy (Pearce et al., 2017), yet, the strength of the association is dependent on the sample studied (Jern et al., 2017; Pearce et al., 2018).

Second, allelic variation in the COMT (catechol-O-methyltransferase) gene might play a role in dopamine signaling because it codes a key enzyme for regulating cortical dopamine release (Tunbridge et al., 2004). The substitution polymorphism of COMT rs4680 has received a lot of attention as the A-allele codes for a methionine instead of a valine amino acid coded for by the G-allele. The A-allele (Met) carriers were shown to have lower COMT enzymatic activity, resulting in higher dopamine levels than individuals with one or two copies of the G-allele (Val) (Stein et al., 2006). Individuals with GG genotype also showed higher empathic concern for an unfortunate other (Ru et al., 2017), higher prosocial behavior (Reuter et al., 2011), and higher scores on a personality dimension, which includes the compassion subscale used in the current study (Baeken et al., 2014). Val carrier “warriors” were further better in the processing of unpleasant stimuli but worse in memory and attention tasks than Met carriers “worriers” (Stein et al., 2006), suggesting that the A-allele might be associated with higher compassion. Two later studies (Calati et al., 2011; Pełka-Wysiecka et al., 2012), however, could not replicate the association of COMT rs4680 with cooperativeness.

A third candidate with known functionality is the DBH (dopamine beta-hydroxylase) gene, which codes the enzyme that converts dopamine to norepinephrine. The T-allele of the *DBH* gene polymorphism rs1611115 is associated with lower plasma DBH activity (Zabetian et al., 2001; Mustapic et al., 2014). Low HBH activity results in lower norepinephrine availability negatively related to several aspects of social behavior, which in turn associates with higher dopamine levels (Marino et al., 2005). The CC genotype of rs1611115 has been related to greater empathic ability (Gong et al., 2014), while the TT genotype is associated with personality traits related to impulsiveness and aggression (Hess et al., 2009).

A functional variable number tandem repeats (VNTR) in the dopamine receptor D4 gene was found to be associated with social cognition (Leerkes et al., 2017) and diverse compassion-related phenotypes (Bachner-Melman et al., 2005; Gong et al., 2014; Uzefovsky et al., 2014). Finally, another VNTR in the dopamine transporter (*SLC6A3)* gene has been further found to be associated with dispositional compassion for others and cooperativeness (Pełka-Wysiecka et al., 2012). Yet, we concentrated the current study on SNPs and excluded other forms of genetic variation.

### The Current Study

The current study examines whether SNPs of known functionality in OXTR, CD38, ANKK1/DRD2, COMT, and DBH are associated with the initial levels of dispositional compassion for others. For the reason that the heritability of closely related phenotypes tends to increase with age (Knafo and Plomin, 2006), comprehensive studies of genetic effects on compassion need to be conducted with longitudinal research designs. Therefore, we also examine the associations of the SNPs with the change in compassion over time. We expect that polymorphisms previously linked to lower oxytocin and higher dopamine levels are associated with lower initial levels of dispositional compassion and a slower increase in compassion over time. These neuropeptides, yet, might work slightly differently in males and females (Benenson, 2014; Carter et al., 2017). Previous studies have found gender-sensitive associations for genetic variation in the oxytocin (Christ et al., 2016; Shang et al., 2017) and dopamine (Pełka-Wysiecka et al., 2012; Uzefovsky et al., 2014) signaling pathways. This suggests that potential differences between male and female participants should be considered when studying molecular genetic associations of compassion. The current study will, therefore, contribute to the ongoing discussion on the etiology and consequences of gender differences in personality traits (Costa et al., 2001; Miettunen et al., 2007; Schmitt et al., 2008) by investigating the underlying neurochemical processes. A well-powered prospective study design is used based on three repeated observations, measured 15 years apart in a representative sample, and assessing compassion with a reliable and well-known inventory.

## Methods

### Procedure and Participants

Participants were drawn from the ongoing Young Finns Study (YFS; Raitakari et al., 2008). The YFS is a population-based, prospective study that, since 1980, follows individuals from six different birth cohorts (3, 6, 9, 12, 15, and 18 years old at the baseline). We used data of the 1980 (T0), 1997 (T1), 2001 (T2), and 2012 (T3) waves. Dispositional compassion for others was measured for the first time when the youngest participants were 20 years old (T1), for a second time 4 years later (T2), and for a third time after another 11 years when the oldest participants were 50 years old (T3).

The original sample consisted of 3,596 individuals, and 2,443 participants were successfully genotyped. In the current study, we excluded those who did not respond to the dependent variable at least once (*n* = 313). The inclusion criteria were met by 2,130 participants (59.2% of the original sample).

The YFS was approved by all participating universities' ethics committees at the beginning of the study in 1980, and the follow-ups were approved by the ethics committee of the University of Turku [vernacular institution name: Varsinais-Suomen sairaanhoitopiirin kuntayhtymä, Eettinen toimikunta, Meeting Number 9/2010; study name, “Lasten sepelvaltimotaudin riskitekijät projekti (Laseri) 30-vuotis seurantatutkimus, 25.8.2010”]. The YFS was conducted in accordance with the Helsinki declaration. Written informed consent was obtained from the participants or their parents if the participant was underage.

### Measures

#### Dispositional Compassion

Dispositional compassion for others was measured with Cloninger's Temperament and Character Inventory (TCI) (Cloninger et al., 1993). Compassion (vs. revengefulness) is a sub-scale of the character trait cooperativeness (Garcia et al., 2017). The scale consists of 10 items [e.g., “It gives me pleasure to see my enemies suffer” (reverse scored); “I hate to see anyone suffer” (positively scored); “It gives me pleasure to help others, even if they have treated me badly” (positively scored); and “I like to imagine my enemies suffering” (reverse scored)]. The questions were answered on a five-point Likert scale. Higher compassion, as measured with the TCI, has been found to be associated with higher social warmth, sociability, and positive emotions (García et al., 2012), better well-being and mental health (Saarinen et al., 2019a,b), and regular healthy behaviors (Gluschkoff et al., 2019). Lower compassion, on the contrary, has been found to be correlated with anger, narcissism, hostility, verbal and physical aggression, while the scales has also discriminant validity (De Fruyt et al., 2006; García et al., 2012). The TCI has demonstrated a stable factorial structure and high internal consistency in the general population as well as in clinical samples (Goncalves and Cloninger, 2010; Vitoratou et al., 2015). The compassion scale has high internal consistency (Cronbach's α_T1−T3_ ≥ 0.86) and a high test–retest reliability (r_T1−>T2_ = 0.60, r_T1−>T3_ = 0.69, both *p* < 0.001). Confirmatory factor analyses confirmed across measurement occasions a good fit of the data to a model that accounts for the correlated error structure between reversely scored items (CFI values ≥ 0.96 and RMSEA values ≤ 0.08). Structural analyses of the TCI compassion (vs. revengefulness) scale can be found in Supplementary S2. The balanced wording of the items further did not have an effect on the below-reported results (for separate analyses for the positively worded compassion and the reversed scored revengefulness items, see Supplementary S3). Descriptive statistics and the missing value pattern of the compassion scale are presented in Table 1.

**Table 1:** Descriptive statistics of the study variables.

	**Year of measurement**	**Participants' age**	**No. of valid answers**	**Missing values**	**Range**	**Median**	**%**
Compassion T1	1997	20–35	1,622	508	1–5	3.60	–
Compassion T2	2001	24–39	1,748	382	1–5	3.80	–
Compassion T3	2012	35–50	1,469	661	1–5	3.80	–
Childhood socioeconomic status	1980	3–18	2,040	90	0–2	0.00	–
Adulthood socioeconomic status	2001 (2012)	24–39 (35–50)	1,875	255	0–2	1.00	–
Male	1980	–	2,130	–	0–1	–	44.0
Year of birth 1977	1980	–	332	–	0–1	–	15.6
Year of birth 1974	1980	–	351	–	0–1	–	16.5
Year of birth 1971	1980	–	367	–	0–1	–	17.2
Year of birth 1968	1980	–	371	–	0–1	–	17.4
Year of birth 1965	1980	–	375	–	0–1	–	17.6
Year of birth 1962	1980	–	335	–	0–1	–	15.7

#### Genotyping and Imputation

The GWAS for the participants of the YFS was performed in 2009 (Smith et al., 2010). Genomic DNA was extracted from peripheral blood leukocytes using a commercially available kit and Qiagen BioRobot M48 Workstation according to the manufacturer's instructions (Qiagen, Hilden, Germany). Genotyping was done for 2,556 samples using custom-built Illumina Human 670 k BeadChip at Welcome Trust Sanger Institute. Genotypes were called using Illuminus clustering algorithm. Fifty-six samples failed Sanger genotyping pipeline QC criteria (i.e., duplicated samples, heterozygosity, low call rate, or Sequenom fingerprint discrepancy). From the remaining 2,500 samples, one sample failed gender check, three were removed due to low genotyping call rate (<0.95), and 54 samples for possible relatedness (pi-hat > 0.2). SNPs (11,766) were excluded based on the Hardy–Weinberg equilibrium test (*p* ≤ 1e−06), 7,746 SNPs failed the missingness test (call rate <0.95), and 34,596 SNPs failed the frequency test (minor allele frequency <0.01). After quality control, 2,443 samples and 546,677 genotyped SNPs remained. Genotype imputation was performed using SHAPEIT v1 for haplotyping, and SNPTEST v2.2.2 and 1000 Genomes Phase I integrated variant set release v3 reference panel for imputation. The imputation quality of 39,346,532 imputed SNPs was good (i.e., squared correlation between imputed and true genotypes ≥ 0.40). We selected SNPs in the oxytocin and dopamine signaling pathways that are known to be functional in humans. ANKK1/DRD2 rs1800497 (T → C) (Noble et al., 1997; Pohjalainen et al., 1998) and COMT rs4680 (G → A) (Stein et al., 2006) were directly genotyped. OXTR rs2268498 (C → T) (Reuter et al., 2017; Zimmermann et al., 2018), CD38 rs3796863 (C → A) (Jin et al., 2007; Feldman et al., 2012), and DBH rs1611115 (T → C) (Zabetian et al., 2001; Mustapic et al., 2014) were imputed. Chromosome, position, and minor allele frequency are indicated in Table 2. Because complex traits like compassion are shaped by many genes of small effect (Manolio et al., 2009), we summed up the assumed risk alleles to form genetic profiles for putatively high dopamine and low oxytocin levels (see Belsky and Israel, 2014).

**Table 2:** Single-nucleotide polymorphisms (SNP) in the oxytocin and dopamine signaling pathways known to be functional in humans.

**SNP**	**Gene**	**Chr**	**Position**	**Risk allele**	**Second allele**	**HWE**	**MAF**	**HWE p-value**	**Imputed**	
rs2268498	OXTR	3	8,812,411	C	Minor	T	Yes	44,1	0.568	Yes
rs3796863	CD38	4	15,849,986	C	Major	A	Yes	36,3	0.708	Yes
rs1800497	ANKK1/DRD2	11	113,270,828	C	Major	T	Yes	20,2	0.233	
rs4680	COMT	22	19,951,271	A	Major	G	Yes	44,6	0.631	
rs1611115	DBH	9	136,500,515	T	Minor	C	Yes	17,3	0.598	Yes

*Chr, chromosome; MAF, minor allele frequency; HWE, Hardy–Weinberg equilibrium. Assumed risk alleles were previously associated with lower oxytocin and higher dopamine levels*.

#### Covariates

The top 10 principal components obtained in a genome-wide association study (GWAS) were included as covariates to account for population stratification (Patterson et al., 2006; Border et al., 2019).

Childhood socioeconomic status (SESC) was assessed when participants were from 3 to 18 years old (1980). SESC was formed based on two indicators: the average of mother's and father's years in education and the annual household income. Adulthood socioeconomic status of the participants (SESA) was assessed with self-reported education and income in 2001 and 2012 (preference was given to the first assessment, if available). Both SES indicators were coded with one point per presence of each of the following: High educational level (tertiary education vs. secondary education or lower) and high income (highest 25% vs. lower). The indicators have a minimum score of 0 (low SES) and a maximum of 2 (high SES).

Other covariates included participant age at baseline (years of birth: 1962, 1965, 1968, 1971, 1974, and 1977) and gender (44.0% men).

### Analyses

Whether selective attrition influenced the results was examined by comparing the included participants with those of the initial sample that were excluded with reference to the study variables by means of chi-squared independence tests and independent sample *t*-tests. Genetic associations were examined utilizing multiple indicators latent growth curve modeling (LGCM) (Bishop et al., 2015; Isiordia and Ferrer, 2018). LGCM allows examining antecedents of individual differences in the initial levels of dispositional compassion for others and the rate of change over time. The combination of repeated measurement occasions and a high number of indicators per occasion maximizes the statistical power of our models (Hertzog et al., 2008; Oertzen et al., 2010). Using LGCM also follows current best practice by accounting for potential measurement error in the phenotype (Border et al., 2019). Furthermore, analyses were conducted with full information maximum likelihood (FIML) estimator to handle missing data (Allison, 2012). FIML estimation does not impute missing values, but it uses all information available, namely, all participants that responded to the compassion scale at least once.

First, we ran a developmental model controlling only for the covariates (Model 1). Second, we entered each of the five functional SNPs separately (Model 2, a–e). Next, we estimated the accumulative effects of genetic profiles for high dopamine (Model 2, f) and low oxytocin (Model 2, g) levels. Forth, we tested for gender-sensitive effects to identify potential differences between men and women in the contribution of allelic variation in the oxytocin and dopamine signaling pathways (Model 3, a–g).

Finally, we conducted several robustness checks. To test the independence of associations, we entered all five functional SNPs jointly (Pearce et al., 2017), which did not alter the main interpretation of the results. The findings were further robust in regard to alternative genetic models (additivity vs. dominance and heterozygotic effects) (Dick et al., 2015). Because some authors argue that the pattern of associations is more important than individual signals for a candidate SNP (e.g., Pearce et al., 2017), we also compared empirically the results for the five functional variants with the performance of additional 10 commonly studied SNPs (see Supplementary).

We used a liberal significance threshold by dividing the *p*-value for nominal significant associations by the number of tested candidate genes, 0.05/5 = 0.01, to account for multiple testing (see Border et al., 2019).

## Results

Attrition analysis indicated that included participants were more likely to be women compared with those participants of the initial sample who were excluded from the current study (56.0 vs. 43.0%; *p* < 0.001). There was no attrition bias in any of the other study variables.

Across models (Table 3), the global fit indices suggested that a linear trajectory fits the data well. RMSEAs of ≤ 0.033 suggested a good fit and CFIs of ≥ 0.913 indicated an acceptable fit.

**Table 3:** Association between oxytocin and dopamine pathway genes and the initial levels of dispositional compassion for others and change over time.

**Model**		**Initial levels**	***SE***	***p***	**Change over time**	***SE***	***p***
***Model 1***	***Developmental model***						
	Latent factors	3.740	0.076	<0.001	0.302	0.049	<0.001
***Model 2***	***Adding the effect of oxytocin and dopamine pathway genes, SNPs entered separately***						
a)	rs2268498 C → T (OXTR)	−0.011	0.034	0.752	−0.009	0.020	0.667
b)	rs3796863 C → A (CD38)	0.012	0.034	0.718	−0.009	0.021	0.684
c)	rs1800497 C → T (ANKK1)	0.016	0.041	0.695	−0.017	0.024	0.477
d)	rs4680 A → G (COMT)	0.008	0.034	0.813	0.007	0.020	0.713
e)	rs1611115 T → C (DBH)	−0.035	0.043	0.427	0.063	0.027	0.018
f)	Low oxytocin genetic profile	0.000	0.014	0.982	0.011	0.024	0.633
g)	High dopamine genetic profile	−0.001	0.023	0.974	0.015	0.014	0.286
***Model 3***	***Women***						
a)	rs2268498 C → T (OXTR)	0.026	0.046	0.571	−0.023	0.026	0.370
b)	rs3796863 C → A (CD38)	0.022	0.045	0.629	−0.002	0.026	0.925
c)	rs1800497 C → T (ANKK1)	−0.024	0.053	0.651	−0.030	0.030	0.317
d)	rs4680 A → G (COMT)	0.011	0.044	0.797	−0.018	0.025	0.474
e)	rs1611115 T → C (DBH)	0.023	0.057	0.681	−0.015	0.032	0.647
f)	Low oxytocin genetic profile	−0.002	0.032	0.010	0.018	0.576
g)	High dopamine genetic profile	−0.006	0.029	0.838	−0.005	0.017	0.756
	***Men***						
	rs2268498 C → T (OXTR)	−0.054	0.051	0.296	0.014	0.034	0.677
	rs3796863 C → A (CD38)	0.006	0.054	0.910	−0.011	0.036	0.751
	rs1800497 C → T (ANKK1)	0.075	0.063	0.234	−0.003	0.042	0.937
	rs4680 A → G (COMT)	−0.046	0.053	0.384	0.018	0.034	0.605
	rs1611115 T → C (DBH)	−0.122	0.068	0.072	0.206	0.046	<0.001
f)	Low oxytocin genetic profile	0.031	0.037	0.408	−0.012	0.024	0.611
g)	High dopamine genetic profile	0.013	0.036	0.710	0.044	0.024	0.064

*In all models (n = 2,130), included covariates were year of birth, gender, childhood, and adulthood socioeconomic status, and the top 10 genetic principal components. Across models, RMSEAs = 0.033 and CFIs = 0.913–0.914. SE = standard error. Statistically significant covariate associations are presented in the main text*.

The developmental model for dispositional compassion for others indicated that initial levels, b = 3.740 (*SE* = 0.076; *p* > 0.001), increased over time, b = 0.302 (*SE* = 0.049; *p* > 0.001) (Model 1). Year of birth 1963, b = 0.255 (*SE* =; *p* = 0.003), and year of birth 1965, b = 0. 178 (*SE* = 0082; *p* = 0.031), were positively associated with a higher initial level of compassion for others. Men, b = −0.215 (SE = 0.050; *p* < 0.001), and participants with lower adulthood SES, b = −0.171 (SE = 0.067; *p* = 0.011), had lower initial levels of compassion. There was also a nominally significant association between the 10th principal component, b = 1.475 (SE = 0.689; *p* = 0.032), and change of dispositional compassion for others over time.

In Model 2, none of the five SNPs with known functionality in the oxytocin and dopamine pathway genes was associated with the initial levels of dispositional compassion for others. However, there was a nominally significant positive association between DBH polymorphism rs1611115 and the change over time of compassion (Model 2e). Individuals who carry one or two copies of the T-allele have increased faster in compassion over time than those homozygotes for the C-allele, b = 0.063 (SE = 0.027; *p* = 0.018). This association, however, did not survive Bonferroni correction for the number of tested candidate genes.

**Figure 1:**
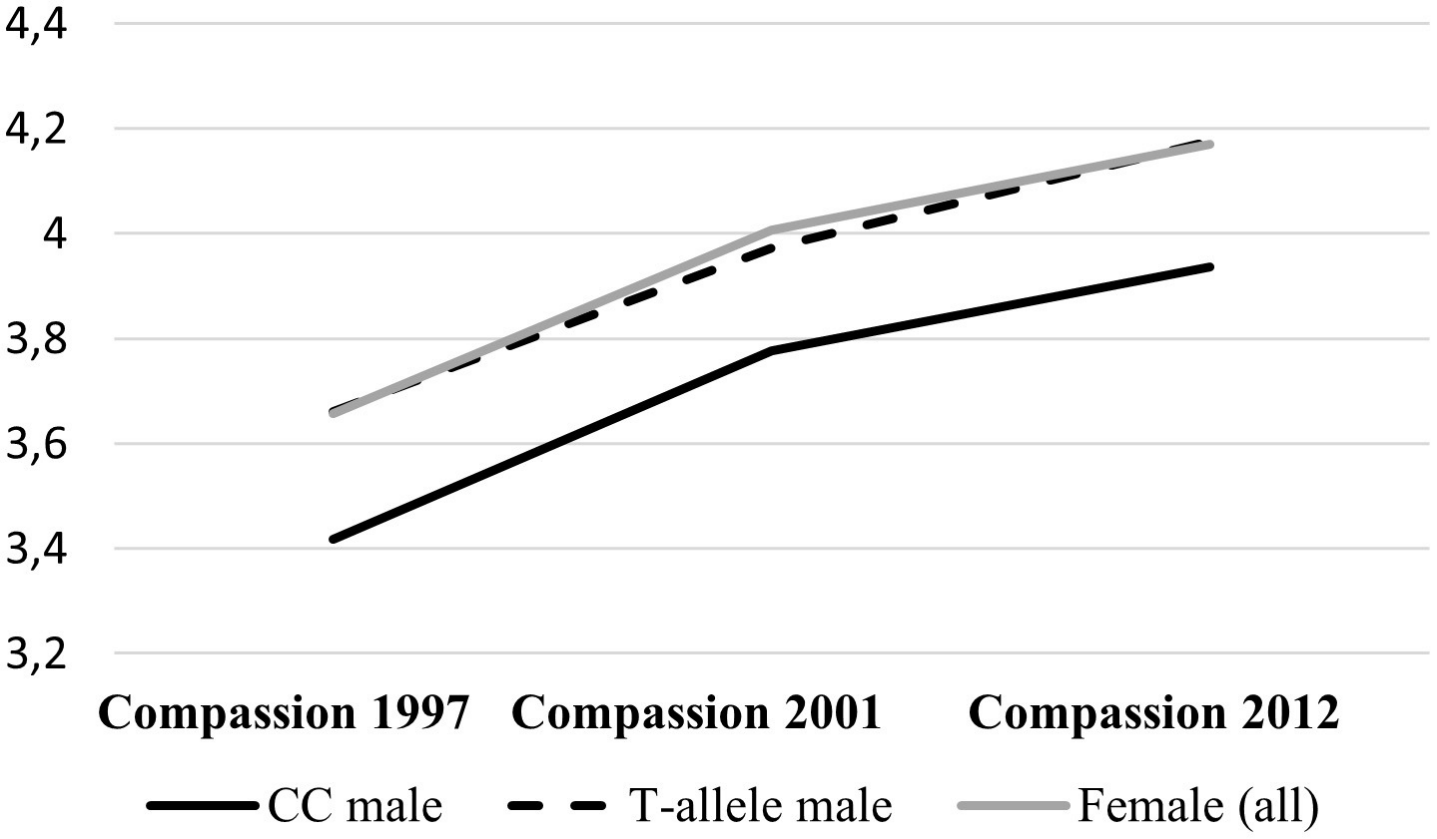
Development of dispositional compassion for others over time. Comparing female participants (*n* = 1,192; gray line) with male participants split by DBH rs1611115 genotype. Continuous black line are CC homozygotes (*n* = 646), and dotted black line are carriers of the T-allele (*n* = 292).

Furthermore, there was a significant interaction between gender and DBH rs1611115 (*p* < 0.001). Separate analyses for male and female participants (Model 3) indicated that the effect of the T-allele on the development of compassion over time was largely driven by men, 0.206 (SE = 0.046; *p* < 0.001), and not significant in women (*p* = 0.647). This male-specific effect remained significant taking into account multiple testing. Figure 1 illustrates this robust gender-sensitive effect of variation in DBH rs1611115. Male carrier of the T-allele (*n* = 292; combined due to the small number of T-allele homozygotes, *n* = 29) showed a similar development of compassion over time compared with women, while CC homozygotes (*n* = 646), on average, reported at all three observations lower dispositional compassion for others.

Finally, neither of the two genetic profiles nor the additional 10 commonly studied SNPs (see Supplementary Table 2) were associated with dispositional compassion.

## Discussion

We examined the association between the development of dispositional compassion for others over the life span and five SNPs previously linked to lower oxytocin (OXTR rs2268498 and CD38 rs3796863) and higher dopamine (ANKK1/DRD2 rs1800497, COMT rs4680, and DBH rs1611115) levels (Marino et al., 2005; Stein et al., 2006; Jin et al., 2007; Feldman et al., 2012). There was no statistically significant association between any of the SNPs and the initial level of compassion. In the dopamine signaling pathway, however, there was a gender-sensitive effect with male participants who carry one or two copies of the T-allele of rs1611115 increasing faster in compassion when growing older compared with those homozygotes for the C-allele. Allelic variation in rs1611115 and changes in compassion over time were not related in female participants when analyzed separately, even though approaching Bonferroni corrected significance levels when men and women were considered together. We further confirmed that men, on average, have lower initial levels of dispositional compassion for others than women (see Hintsanen et al., 2019). Taken together, these findings imply that men may leap up from this lower starting point if they carry the T-allele of DBH rs1611115.

Also, two previous studies found gender-sensitive associations for dopamine pathway genes (Pełka-Wysiecka et al., 2012; Uzefovsky et al., 2014). We speculate that the found male-specific effects could be a joint product of genes and environments. That is, males with specific alleles are more likely to end up in different environments and to develop a higher level of compassion due to the environmental circumstances (see Nettle, 2006; Abramson et al., 2020). This might happen due to well-documented gender differences in personality traits, such as assertiveness or reward dependence (Costa et al., 2001; Miettunen et al., 2007), which are found to be larger in developed and egalitarian countries in which women have more equal opportunities such as Finland (Schmitt et al., 2008). Certainly, there also is a tendency in humans to become more compassionate as we grow older because of aging-related role changes, such as fewer demands of competing for social place, and life events, such as the loss of a beloved person (Cuddy et al., 2005; Hintsanen et al., 2019). The found association between the genetic variation in the dopamine pathway and change in compassion over time is in line with studies that suggest that dopamine signaling is neurologically linked to applying “warrior” over “worrier” strategies (Stein et al., 2006). That this effect was gendered and stronger in male participants implies that functions of the studied neuropeptides are critical for gender differences in compassion development (e.g., Carter et al., 2017).

That we did not find any relationships of dispositional compassion with neither four out of five functional SNPs nor the two genetic profiles conflicts with previous work on compassion-related traits and states (Rodrigues et al., 2009; Gong et al., 2014; Christ et al., 2016; Liu et al., 2017; Pearce et al., 2017; Ru et al., 2017). At the same time, the reviewed empirical literature is heterogeneous, including more null findings for included SNPs than the highlighted significant associations (Comings et al., 2001; Pełka-Wysiecka et al., 2012; Liu et al., 2017; Pearce et al., 2017) and associations pointing into the opposite direction (Laursen et al., 2014). This pairs with general criticism of the candidate gene approach because associations with individual SNPs often cannot be replicated (Hewitt, 2012; Border et al., 2019). The complexity of prosocial behavior also requires neurological mechanisms to protect oneself and close others from potentially threatening individuals or groups (Carter et al., 2017). The role of oxytocin has been implicated in the punishment of those who caused the suffering of a close other and increased ethnocentrism (De Dreu et al., 2011; Pfattheicher et al., 2019). This context dependence might be another reason why we did not find significant associations between compassion and oxytocin pathway genes. Also available GWAS in phenotypes loosely related to compassion did not find associations for any of the suggested candidate genes (Warrier et al., 2018a,b). Another group of researchers has argued that associations for candidate genes are dependent on the social domain under study (Li et al., 2015; Gong et al., 2017; Pearce et al., 2017). In the latter view, the current study presents a novel association. No previous study with comparable statistical power (see Pełka-Wysiecka et al., 2012) has examined the contribution of functional variants in oxytocin and dopamine pathway genes on dispositional compassion for others.

The reversed directional association—contrary to previous work (Hess et al., 2009; Gong et al., 2014)—indicates that higher, and not lower, dopamine levels relate to higher compassion. There are several explanations for this unexpected finding. Oxytocin and dopamine are known to interact (Baskerville and Douglas, 2010; Feldman et al., 2012). Oxytocin release notably increases collaboration between people and prosocial behavior, which in turn might activate the dopaminergic reward centers of the brain (Skuse and Gallagher, 2009). In line with this reasoning, Inagaki et al. (2016) have found that giving social support rather than receiving it is associated with higher well-being, reduced stress-related brain activity, and greater reward-related activity. There further exists a link between compassionate states and dopaminergic reward signaling (Kim et al., 2009). Being compassionate has also been found to associate with better health, psychological well-being, and social functioning (Post, 2005; Steffen and Masters, 2005; Saarinen et al., 2019b). Dispositional compassion for others further predicts higher positive affect, lower negative affect, and more perceived social support assessed 4 and 10 years later (Saarinen et al., 2019b).

Moreover, these putatively higher dopamine levels are a consequence of reduced plasma DBH enzyme activity when converting dopamine to norepinephrine (Zabetian et al., 2001; Marino et al., 2005; Mustapic et al., 2014). Thus, allelic variation based on dopamine signaling activity rather than overall levels may be important for compassion development (Stein et al., 2006; Nikolova et al., 2011). The found association between dopamine signaling activity and compassion is interesting on its own right by illuminating a specific genetic pathway that might explain individual differences in compassion for others. It is also important for informing subsequent gene–environment interaction studies and meta-analyses.

That we did not find significant associations for any of the additionally tested 10 SNPs was somewhat surprising because phenotypes closely related to compassion for others such as empathic concern (Huetter et al., 2016), autonomic arousal while witnessing others suffering (Smith et al., 2014), and physiological and self-reported stress reactivity (Rodrigues et al., 2009) were previously associated with these SNPs. These null findings indicate that there may be differences in the genetic background of dispositional vs. state-like compassion.

### Limitations and Strengths

The current study has limitations that need to be considered when interpreting the results. That selective attrition in terms of gender influenced the results limits the generalizability of our findings, especially, because the positive effect of carrying the T-allele of DBH rs1611115 on dispositional compassion for others was stronger in men, while women were somewhat overrepresented. The study was conducted in a Caucasian population, and the findings might not apply in other cultures even though compassion is stated to be a universal disposition (Cloninger et al., 1993; Goetz et al., 2010).

For the OXTR, the second functional SNP rs35062132 (Ma et al., 2013) is, unfortunately, monomorphic in Finnish samples and could not be included in our study. Finally, we cannot rule out the possibility that there might be other candidate genes and genetic variation, such as VNTRs, that contribute to individual differences in compassion (see Pełka-Wysiecka et al., 2012).

Our data only allows to study the development of compassion in individuals between age 20 and 50. It would be interesting to know what happens when the participants grow even older. We are also unable to say if there were differences in the level of compassion due to allelic variation when the participants were younger than in our assessments.

There is a lack of consensus on the definition and measurement of compassion (Goetz et al., 2010; Strauss et al., 2016; Pommier et al., 2019). Using a reliable and validated personality inventory enabled us, contrary to most previous studies, to investigate molecular genetic underpinnings of dispositional compassion for others (Cloninger et al., 1993; Garcia et al., 2017). The focus of this study was on the stable motivational component of compassion, while we did not measure the emotional component or compassionate actions.

Candidate gene approaches have been strongly criticized recently, and current results need to be interpreted with caution pending replication (Hewitt, 2012). Very few, if any, genetically informed cohorts include Cloninger's TCI and have a similar long follow-up, which could be used in replication efforts. We followed the recommendations of Dick et al. (2015) and Border et al. (2019) for reducing the chance of reporting false-positive associations and conducted an extensive set of robustness checks. We achieved sufficient statistical power to detect even small effects by using a longitudinal research design with multiple indicators for measuring compassion for others (Hertzog et al., 2008; Oertzen et al., 2010).

## Conclusions

Genetic variation, previously linked to lower oxytocin and higher dopamine levels, was not related to lower dispositional compassion and a slower increase in compassion over time when male and female participants were analyzed jointly. However, males who were shown to have lower average levels of compassion than women seem to reduce this difference to some degree over time if they carry the T-allele of DBH polymorphism rs1611115 and not the CC genotype. The male-specific finding suggests that gender differences should be paid more attention to in future research. The direction of the association further indicates that dopamine signaling activity rather than overall dopamine levels might drive differences in dispositional compassion.

## Data Availability Statement

The dataset supporting the conclusions of this article was obtained from the Cardiovascular Risk In Young Finns Study (YFS) which comprises health-related participant data. The use of data is restricted under the regulations on professional secrecy (Act on the Openness of Government Activities, 612/1999) and on sensitive personal data (Personal Data Act, 523/1999, implementing the EU data protection directive 95/46/EC). Due to these restrictions, the data cannot be stored in public repositories or otherwise made publicly available. Data access may be permitted on a case-by-case basis upon request only. Data sharing outside the group is done in collaboration with the YFS group and requires a data-sharing agreement. Investigators can submit an expression of interest to the chairman of the publication committee (Prof. Mika Kähönen, Tampere University, Finland).

## Ethics Statement

The studies involving human participants were reviewed and approved by all participating universities' ethics committees at the beginning of the study in 1980, and the followups were approved by the ethics committee of the University of Turku (vernacular institution name: Varsinais-Suomen sairaanhoitopiirin kuntayhtymä, Eettinen toimikunta, Meeting Number 9/2010; study name, Lasten sepelvaltimotaudin riskitekijät projekti (Laseri) 30-vuotis seurantatutkimus, 25.8.2010). The YFS was conducted in accordance with the Helsinki declaration. Written informed consent was obtained from the participants. Written informed consent to participate in this study was provided by the participants' legal guardian/next of kin.

## Author Contributions

All the authors have made a substantial contribution to designing or carrying out the research, writing or revising the manuscript, or providing guidance on the execution of the research. All analyses have been conducted by HD. The paper has been read and approved by all the authors.

## Conflict of Interest

L-PL and TL are employed by the company Fimlab Laboratories Oy. The remaining authors declare that the research was conducted in the absence of any commercial or financial relationships that could be construed as a potential conflict of interest.
