# Supplementary material for: Functional Polymorphisms in Oxytocin and Dopamine Pathway Genes and the Development of Dispositional Compassion Over Time: The Young Finns Study
Source: Front Psychol. 2021 Apr 8;12:576346. doi: 10.3389/fpsyg.2021.576346 (PMC8060576; doi:10.3389/fpsyg.2021.576346)
Supplement: Supplementary file 1 [file Data_Sheet_1.docx]

**Supplement to “Functional polymorphisms in oxytocin and dopamine pathway genes and the development of dispositional compassion over time: The Young Finns Study”**

*S1) Testing additional SNPs commonly used in the literature.*

We additionally selected two commonly studied SNPs for each of the genes of interest which are, if possible, maximally informative for a set of SNPs (tagSNPs) or haplotype tagging (htSNPs). Variants rs53576 (G🡪A) and rs2254298 (A🡪G) of OXTR, rs6449182 (G🡪C) and rs12644506 (T🡪C) of CD38, rs1801028 (C🡪G) and rs468317 (G🡪A) ANKK1/DRD2; rs4633 (T🡪C) and rs4818 (G🡪C) of COMT, and rs2519152 (C🡪T) and rs6271 (T🡪C) of DBH. This allows us to compare the pattern of association of functional versus commonly studies variants. Chromosome, position, and minor allele frequency of these ten SPNs are presented in Table S1, with the genotype frequencies following the Hardy-Weinberg Equilibrium taken into account the number of conducted tests (p ≥ .01). The Table also presents the assumed risk allele of the SNPs, if the functionality is known, and whether it was directly genotyped or imputed.

*Oxytocin pathway genes*. The G-allele OXTR rs53576 was meta-analytically related to empathy (Gong et al., 2017). Neuro-scientific studies have shown that carrying the A-allele (versus the GG genotype) was associated to lower hypothalamus volume in facial emotion recognition tasks and an increase in the structural hypothalamus-amygdala coupling (Tost et al., 2010). Another OXTR variant that was associated with individual differences in oxytocin release is rs2254298 in a way that GG homozygotes had lower plasma oxytocin levels compared to A-allele carriers (Feldman et al., 2012). And allelic variation in this SNP broadly associates with structural and functional changes in oxytocinergic brain regions (Furman, Chen, & Gotlib, 2011; Inoue et al., 2010; Tost et al., 2011). As part of a genic profile, OXTR rs2254298 predicted communicative empathy (Schneiderman, Kanat-Maymon, Ebstein, & Feldman, 2014) and parental sensitivity (Feldman et al., 2012).

Allelic variation CD38 rs6449182 has not yet been examined in relation to compassion-related phenotypes but carrying the G-allele was associated with a significantly reduced expression of CD38 (Riebold et al., 2011). The T-allele of CD38 variant rs12644506 (C to T) was further associated with less social connectedness (Chang et al., 2014).

We have further excluded several OXTR SNPs that were previously studied in relation to compassion related phenotypes, such as perspective-taking and empathic concern (Christ, Carlo, & Stoltenberg, 2016; Wu, Li, & Su, 2012), to balance the number of analyses that we ran for each of the five candidate genes.

*Dopamine pathway genes*. For DRD2 SNP rs1801028 it is known that the C-allele codes for the amino acid serine which is substituted by cysteine for carriers of the G-allele, while also the C-allele of rs468317 has been studied as a risk allele in relation to self-reported empathy (Glatt & Jönsson, 2006; Pearce, Wlodarski, Machin, & Dunbar, 2017).

Allelic variation in rs4633 and rs4818 do not change the amino acid sequence of the COMT protein, yet, carrying the C-allele has been associated with various socio-emotional phenotypes (Kocabas et al., 2010).

Even though the largest proportion of the human DBH activity is explained by variant rs1611115, two more SNPs in the DBH gene (rs2519152 and rs6271) were found to have incremental effects (Tang, Epstein, Anderson, Zabetian, & Cubells, 2007).

**Table S1.** Commonly Studied Single Nucleotide Polymorphisms (SNP) in the Oxytocin and Dopamine Signaling Pathway

| SNP | Gene | Chr | Position | Coded allele | | Second allele | Assumed risk allele | Funct-ional | MAF | HWE p-value | Imputed |
| --- | --- | --- | --- | --- | --- | --- | --- | --- | --- | --- | --- |
| rs53576 | OXTR | 3 | 8804371 | A | (Minor) | G | A |  | 42,7 | 0.019 |  |
| rs2254298 | OXTR | 3 | 8802228 | G | (Major) | T | T |  | 8,6 | 0.584 | Yes |
| rs6449182 | CD38 | 4 | 15780453 | C | (Major) | G | G |  | 21,2 | 0.272 | Yes |
| rs12644506 | CD38 | 4 | 15799878 | C | (Major) | T | T |  | 14 | 0.331 | Yes |
| rs1801028 | DRD2 | 11 | 113283484 | G | (Major) | C | G | Yes | 21,6 | 1.000 | Yes |
| rs468317 | DRD2 | 21 | 19131877 | T | (Minor) | C | C |  | 22,5 | 0.102 | Yes |
| rs4633 | COMT | 22 | 19950235 | C | (Minor) | T | T |  | 44,7 | 0.694 | Yes |
| rs4818 | COMT | 22 | 19951207 | C | (Major) | G | G |  | 30,6 | 0.224 | Yes |
| rs2519152 | DBH | 9 | 136509634 | T | (Major) | C | T |  | 42,7 | 0.248 | Yes |
| rs6271 | DBH | 9 | 136522274 | C | (Major) | T | C |  | 7,7 | 0.029 | Yes |

*Notes.* Chr = Chromosome, MAF = Minor allele frequency, HWE = Hardy-Weinberg Equilibrium

| **Table S2.** Robustness Checks by Repeating the Analyses with Commonly Studied Single Nucleotide Polymorphisms (SNP) | | | | | | | | |
| --- | --- | --- | --- | --- | --- | --- | --- | --- |
|  | *Initial levels* | *(SE)* | *p* |  | Change over time | *(SE)* | *p* |  |
|  |  |  |  |  |  |  |  |  |
| rs53576 (OXTR) | 0.016 | 0.033 | .621 |  | 0.003 | 0.020 | .878 |  |
| rs2254298 (OXTR) | 0.011 | 0.058 | .850 |  | 0.017 | 0.036 | .634 |  |
| rs6449182 (CD38) | 0.050 | 0.040 | .208 |  | -0.037 | 0.024 | .131 |  |
| rs12644506 (CD38) | 0.026 | 0.047 | .579 |  | 0.004 | 0.028 | .894 |  |
| rs1801028 (DRD2) | 0.115 | 0.117 | .325 |  | 0.018 | 0.073 | .804 |  |
| rs468317 (DRD2) | 0.068 | 0.040 | .086 |  | 0.024 | 0.024 | .325 |  |
| rs4633 (COMT) | 0.007 | 0.033 | .827 |  | 0.006 | 0.020 | .749 |  |
| rs4818 (COMT) | 0.033 | 0.035 | .351 |  | 0.012 | 0.021 | .584 |  |
| rs2519152 (DBH) | 0.031 | 0.034 | .361 |  | 0.031 | 0.021 | .142 |  |
| rs6271 (DBH) | 0.019 | 0.068 | .780 |  | 0.032 | 0.041 | .444 |  |

Notes. In all models, included covariates were year of birth, gender, childhood and adulthood socioeconomic status, and the top 10 genetic principal components. Across models, RMSEAs = 0.033 and CFIs = 0.913

S2) *Factor structure of the TCI Compassion (vs. Revengefulness) scale.*

We used YFS data from 1997 (T1), when participants were from 20 to 35 years old (n= 2130), to assess the factor structure of the TCI compassion (vs. revengefulness) scale. The five items measuring revengefulness have been reversely scored before the analyses.

A confirmatory factor analysis (CFA) with all ten items loading on a single latent factor (Figure S1) resulted in a rather poor fit to the data (RMSEA = 0.119 / CFI = 0.880). The BIC was 40178.84.

When modeling two separate oblique factors of compassion and revengefulness (Figure S2) the model fit improved (RMSEA = 0.077 / CFI = 0.951). BIC reduced to 39712.50, indicating a better fit of this model to the data than for the previous model. Unlike in other compassion scales and samples (Keller & Pfattheicher, 2013), there was no negative relationship between the compassion factor and the factor including antisocial elements (*r* = .39, after item reversal).

However, two separate factors are not the proposed factorial structure for the TCI subscales (Cloninger, Svrakic, & Przybeck, 1993; Goncalves & Cloninger, 2010; Vitoratou, Ntzoufras, Theleritis, Smyrnis, & Stefanis, 2015). Thus, we run a third CFA. The final single-factor model of TCI compassion (vs. revengefulness) with correlated residuals (Figure S3), used in the main analyses, that accounts for the correlated error structure between the reversely scored (revengefulness) items had a very good fit to the data (RMSEA = 0.060 / CFI = 0.978). The best-fitting model with correlated errors between the reversely scored items, BIC = 39592.79, assumes that individual differences in response styles (Biderman, McAbee, Job Chen, & Hendy, 2018) and promotion versus prevention focus (Higgins, 1998) should be accounted for when modeling a balanced scale such as TCI Compassion (vs. Revengefulness).

Finally, we constraint the factor loadings to be equal across gender in a multigroup CFA to see whether there are differences between men and women. A chi-square test was used to compare the constraint (RMSEA = 0.055 / CFI = 0.978) to the unconstraint model. A p-value of 0.713 indicated that the model fit did not decrease significantly due to differences in the factor loadings between gender.


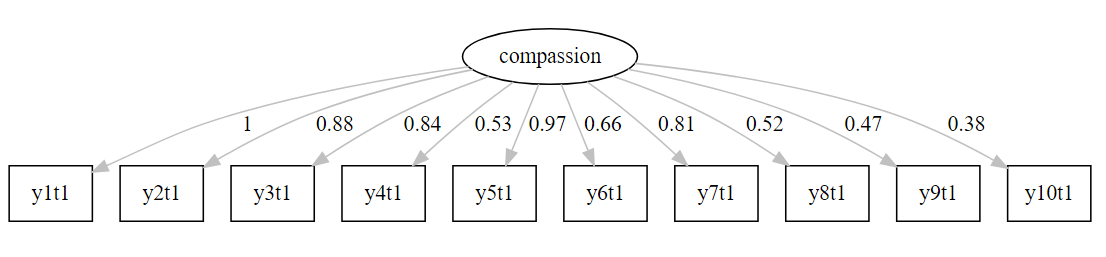


Figure S1: One factor model of TCI compassion (vs. revengefulness)


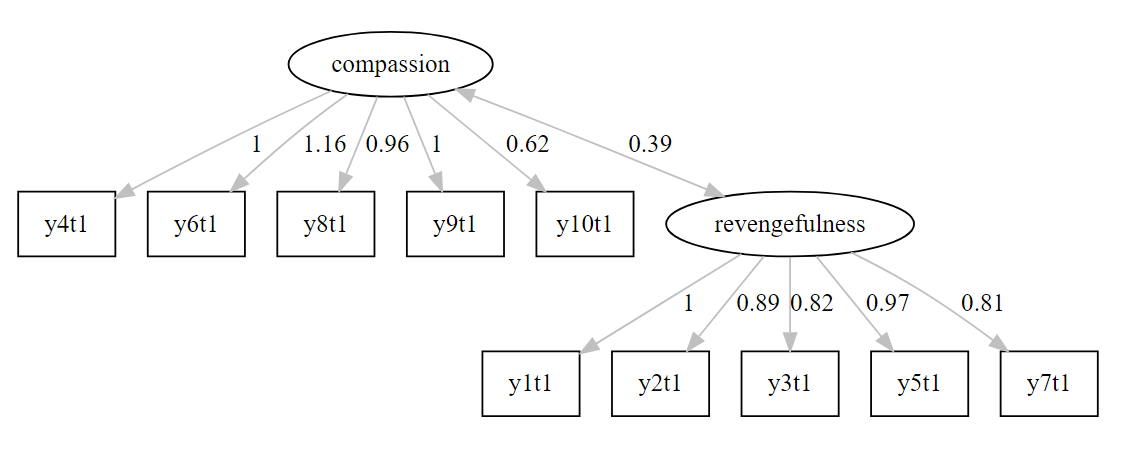
 Figure S2: Two factor model of compassion and revengefulness modeled separately


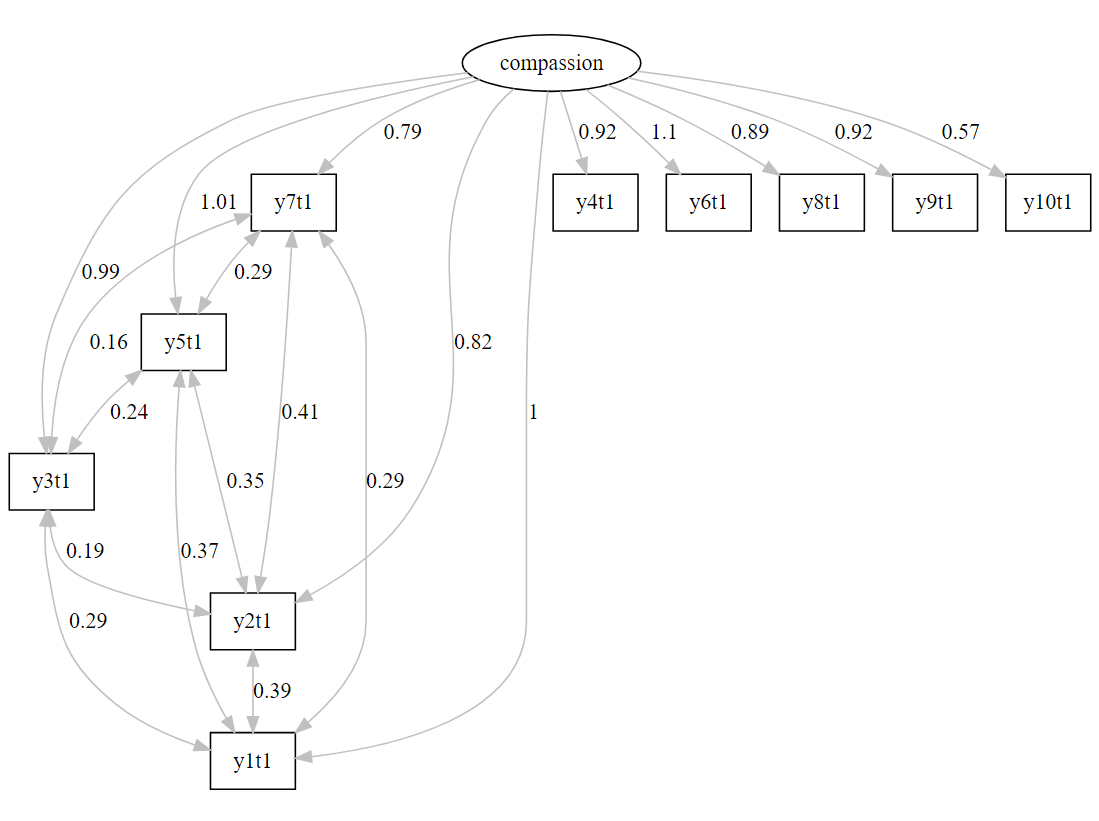


Figure S3: The final one-factor model of TCI compassion (vs. revengefulness) with correlated residuals that accounts for the correlated error structure between reversely scored items

*S3) Robustness test of the effect of item wording on oxytocin and dopamine function*

Separate multiple indicators latent growth curve models using only positively scored items (compassion; RMSEA < 0.040 / CFI > 0. 880) versus reversed items (revengefulness; RMSEA < 0.030 / CFI > 0.960) were conducted. The data in Tables S2 and S3 showed that the change in compassion (vs. revengefulness) over time was mostly driven by the reversely scored items, b = 0.281 (SE = 0.051; p < .001), and that the slope was not significant in positively scored items.

In both models, based on positively scored items, b = 0.059 (SE = 0.027; p = .029), and reversely scored items, b = 0.057 (SE = 0.029; p = .045), a similar pattern of genetic effects was found. In line with the results reported in the main paper, the found associations suggest that compassion increased faster over time if individuals carry the T-allele of rs1611115. In the model for reversed items there were further two new associations for the slope and rs4680, b = 0.044 (SE = 0.022; p = .041), and high dopamine genetic profile, b = 0.029 (SE = 0.015; p = .050). None of these effects, however, were strong enough to survive correction for multiple testing.

The effect of rs1611115 was not moderated by gender when analyzing positively scored items and reversely scored items separately. Like in the main analyses, we did not find any association in female participants. In male participants, however, the genetic effect on the slop of compassion was found in positively scored items, b = 0.188 (SE = 0.047; p < .001), as well as in reversed items, b = 0.140 (SE = 0.049; p = .004).

**Table S3.** Association between Oxytocin and Dopamine Pathway Genes and a Model using only the Positively Scored TCI Items (High Compassion)

| *Model* |  | Initial levels | *(SE)* | *p* |  | Change over time | *(SE)* | *p* |
| --- | --- | --- | --- | --- | --- | --- | --- | --- |
| *Model 1* | *Developmental model* | | | | | | | |
|  | Latent factors | 3.419 | 0.076 | **<.001** |  | 0.008 | 0.048 | 0.868 |
|  |  |  |  |  |  |  |  |  |
| *Model 2* | *Adding the effect of oxytocin and dopamine pathway genes, SNPs entered separately* | | | | | | | |
| a) | rs2268498 C🡪T (OXTR) | -0.005 | 0.034 | .879 |  | 0.007 | 0.021 | .729 |
| b) | rs3796863 C🡪A (CD38) | 0.001 | 0.035 | .987 |  | -0.018 | 0.021 | .396 |
| c) | rs1800497 C 🡪 T (ANKK1) | 0.041 | 0.041 | .315 |  | -0.009 | 0.025 | .704 |
| d) | rs4680 A 🡪 G (COMT) | 0.022 | 0.034 | .516 |  | -0.016 | 0.021 | .445 |
| e) | rs1611115 T🡪C (DBH) | -0.039 | 0.044 | .374 |  | 0.059 | 0.027 | **.029** |
| f) | low oxytocin genetic profile | -0.002 | 0.024 | .921 |  | -0.005 | 0.015 | .733 |
| g) | high dopamine genetic profile | 0.012 | 0.023 | .592 |  | 0.006 | 0.014 | .691 |
|  |  |  |  |  |  |  |  |  |
| *Model 3* | *Women* | | | | | | | |
| a) | rs2268498 C🡪T (OXTR) | -0.045 | 0.046 | .330 |  | 0.031 | 0.026 | .229 |
| b) | rs3796863 C🡪A (CD38) | -0.008 | 0.045 | .866 |  | 0.007 | 0.026 | .793 |
| c) | rs1800497 C 🡪 T (ANKK1) | 0.016 | 0.054 | .763 |  | -0.038 | 0.030 | .215 |
| d) | rs4680 A 🡪 G (COMT) | -0.008 | 0.044 | .860 |  | -0.000 | 0.025 | .991 |
| e) | rs1611115 T🡪C (DBH) | 0.012 | 0.057 | .835 |  | -0.010 | 0.032 | .765 |
| f) | low oxytocin genetic profile | -0.026 | 0.032 | .424 |  | 0.018 | 0.018 | .306 |
| g) | high dopamine genetic profile | 0.005 | 0.029 | .873 |  | -0.014 | 0.017 | .398 |
|  |  |  |  |  |  |  |  |  |
|  | *Men* | | | | | | | |
|  | rs2268498 C🡪T (OXTR) | 0.042 | 0.052 | .420 |  | -0.028 | 0.034 | .413 |
|  | rs3796863 C🡪A (CD38) | 0.023 | 0.055 | .679 |  | -0.054 | 0.036 | .138 |
|  | rs1800497 C 🡪 T (ANKK1) | 0.078 | 0.064 | .224 |  | 0.028 | 0.043 | .515 |
|  | rs4680 A 🡪 G (COMT) | 0.072 | 0.053 | .176 |  | -0.045 | 0.035 | .196 |
|  | rs1611115 T🡪C (DBH) | -0.120 | 0.069 | .083 |  | 0.188 | 0.047 | **<.001** |
| f) | low oxytocin genetic profile | 0.032 | 0.038 | .395 |  | -0.039 | 0.025 | .112 |
| g) | high dopamine genetic profile | 0.028 | 0.036 | .448 |  | 0.036 | 0.024 | .133 |

*Notes*. In all models (n=2130), included covariates were year of birth, gender, childhood and adulthood socioeconomic status, and the top 10 genetic principal components. Across models, RMSEAs = 0.032-0.035 and CFIs = 0.882-0.894.

**Table S4.** Association between Oxytocin and Dopamine Pathway Genes and a Model using only the Reversed Scored TCI Items (Low Revengefulness)

| *Model* |  | Initial levels | *(SE)* | *p* |  | Change over time | *(SE)* | *p* |
| --- | --- | --- | --- | --- | --- | --- | --- | --- |
| *Model 1* | *Developmental model* | | | | | | | |
|  | Latent factors | 3.651 | 0.074 | **<.001** |  | 0.281 | 0.051 | **<.001** |
|  |  |  |  |  |  |  |  |  |
| *Model 2* | *Adding the effect of oxytocin and dopamine pathway genes, SNPs entered separately* | | | | | | | |
| a) | rs2268498 C🡪T (OXTR) | 0.033 | 0.033 | .323 |  | -0.007 | 0.022 | .764 |
| b) | rs3796863 C🡪A (CD38) | 0.012 | 0.034 | .730 |  | 0.017 | 0.022 | .437 |
| c) | rs1800497 C 🡪 T (ANKK1) | -0.005 | 0.040 | .907 |  | -0.021 | 0.026 | .426 |
| d) | rs4680 A 🡪 G (COMT) | -0.028 | 0.033 | .390 |  | 0.044 | 0.022 | **.041** |
| e) | rs1611115 T🡪C (DBH) | -0.033 | 0.042 | .431 |  | 0.057 | 0.028 | **.045** |
| f) | low oxytocin genetic profile | 0.022 | 0.023 | .345 |  | 0.005 | 0.015 | .745 |
| g) | high dopamine genetic profile | -0.023 | 0.022 | .296 |  | 0.029 | 0.015 | **.050** |
|  |  |  |  |  |  |  |  |  |
| *Model 3* | *Women* | | | | | | | |
| a) | rs2268498 C🡪T (OXTR) | 0.002 | 0.045 | .964 |  | -0.001 | 0.028 | .963 |
| b) | rs3796863 C🡪A (CD38) | 0.038 | 0.044 | .391 |  | -0.001 | 0.028 | .979 |
| c) | rs1800497 C 🡪 T (ANKK1) | -0.060 | 0.052 | .252 |  | -0.013 | 0.033 | .688 |
| d) | rs4680 A 🡪 G (COMT) | -0.040 | 0.043 | .349 |  | 0.047 | 0.027 | .085 |
| e) | rs1611115 T🡪C (DBH) | 0.005 | 0.056 | .925 |  | 0.004 | 0.035 | .917 |
| f) | low oxytocin genetic profile | 0.020 | 0.031 | .523 |  | -0.001 | 0.020 | .957 |
| g) | high dopamine genetic profile | -0.034 | 0.029 | .228 |  | 0.018 | 0.018 | .327 |
|  |  |  |  |  |  |  |  |  |
|  | *Men* | | | | | | | |
|  | rs2268498 C🡪T (OXTR) | 0.064 | 0.050 | .201 |  | -0.009 | 0.035 | .792 |
|  | rs3796863 C🡪A (CD38) | -0.024 | 0.052 | .650 |  | 0.058 | 0.037 | .122 |
|  | rs1800497 C 🡪 T (ANKK1) | 0.070 | 0.062 | .254 |  | -0.033 | 0.044 | .453 |
|  | rs4680 A 🡪 G (COMT) | -0.019 | 0.051 | .714 |  | 0.046 | 0.036 | .195 |
|  | rs1611115 T🡪C (DBH) | -0.076 | 0.066 | .249 |  | 0.140 | 0.049 | **.004** |
| f) | low oxytocin genetic profile | 0.023 | 0.036 | .533 |  | 0.022 | 0.025 | .392 |
| g) | high dopamine genetic profile | -0.006 | 0.035 | .858 |  | 0.048 | 0.025 | .053 |

*Notes*. In all models (n= 2130), included covariates were year of birth, gender, childhood and adulthood socioeconomic status, and the top 10 genetic principal components. Across models, RMSEAs = 0.028-0.029 and CFIs = 0.960-0.962.

**References**

Biderman, M. D., McAbee, S. T., Job Chen, Z., & Hendy, N. T. (2018). Assessing the evaluative content of personality questionnaires using bifactor models. *Journal of Personality Assessment*, *100*(4), 375–388. https://doi.org/10.1080/00223891.2017.1406362

Chang, S.-C., Glymour, M. M., Rewak, M., Cornelis, M. C., Walter, S., Koenen, K. C., … Kubzansky, L. D. (2014). Are genetic variations in OXTR, AVPR1A, and CD38 genes important to social integration? Results from two large U.S. cohorts. *Psychoneuroendocrinology*, *39*, 257–268. https://doi.org/10.1016/J.PSYNEUEN.2013.09.024

Christ, C. C., Carlo, G., & Stoltenberg, S. F. (2016). Oxytocin receptor ( OXTR ) single nucleotide polymorphisms indirectly predict prosocial behavior through perspective taking and empathic concern. *Journal of Personality*, *84*(2), 204–213. https://doi.org/10.1111/jopy.12152

Cloninger, C. R., Svrakic, D. M., & Przybeck, T. R. (1993). A psychobiological model of temperament and character. *Archives of General Psychiatry*, *50*(12), 975–990. https://doi.org/10.1001/archpsyc.1993.01820240059008

Feldman, R., Zagoory-Sharon, O., Weisman, O., Schneiderman, I., Gordon, I., Maoz, R., … Ebstein, R. P. (2012). Sensitive parenting is associated with plasma oxytocin and polymorphisms in the OXTR and CD38 genes. *Biological Psychiatry*, *72*(3), 175–181. https://doi.org/10.1016/J.BIOPSYCH.2011.12.025

Furman, D. J., Chen, M. C., & Gotlib, I. H. (2011). Variant in oxytocin receptor gene is associated with amygdala volume. *Psychoneuroendocrinology*, *36*(6), 891–897. https://doi.org/10.1016/j.psyneuen.2010.12.004

Glatt, S. J., & Jönsson, E. G. (2006). The Cys allele of theDRD2 Ser311Cys polymorphism has a dominant effect on risk for schizophrenia: Evidence from fixed- and random-effects meta-analyses. *American Journal of Medical Genetics Part B: Neuropsychiatric Genetics*, *141B*(2), 149–154. https://doi.org/10.1002/ajmg.b.30273

Goncalves, D. M., & Cloninger, C. R. (2010). Validation and normative studies of the Brazilian Portuguese and American versions of the Temperament and Character Inventory - Revised (TCI-R). *Journal of Affective Disorders*, *124*(1–2), 126–133. https://doi.org/10.1016/j.jad.2009.11.007

Gong, P., Fan, H., Liu, J., Yang, X., Zhang, K., & Zhou, X. (2017). Revisiting the impact of OXTR rs53576 on empathy: A population-based study and a meta-analysis. *Psychoneuroendocrinology*, *80*, 131–136. https://doi.org/10.1016/J.PSYNEUEN.2017.03.005

Higgins, E. T. (1998). Promotion and prevention: Regulatory focus as a motivational principle. *Advances in Experimental Social Psychology*, *30*(C), 1–46. https://doi.org/10.1016/S0065-2601(08)60381-0

Inoue, H., Yamasue, H., Tochigi, M., Abe, O., Liu, X., Kawamura, Y., … Kasai, K. (2010). Association between the oxytocin receptor gene and amygdalar volume in healthy adults. *Biological Psychiatry*, *68*(11), 1066–1072. https://doi.org/10.1016/J.BIOPSYCH.2010.07.019

Keller, J., & Pfattheicher, S. (2013). The compassion-hostility paradox: The interplay of vigilant, prevention-focused self-regulation, compassion, and hostility. *Personality and Social Psychology Bulletin*, *39*(11), 1518–1529. https://doi.org/10.1177/0146167213499024

Kocabas, N. A., Faghel, C., Barreto, M., Kasper, S., Linotte, S., Mendlewicz, J., … Massat, I. (2010). The impact of catechol-O-methyltransferase SNPs and haplotypes on treatment response phenotypes in major depressive disorder: A case–control association study. *International Clinical Psychopharmacology*, *25*(4), 218–227. https://doi.org/10.1097/YIC.0b013e328338b884

Pearce, E., Wlodarski, R., Machin, A., & Dunbar, R. I. M. (2017). Variation in the β-endorphin, oxytocin, and dopamine receptor genes is associated with different dimensions of human sociality. *Proceedings of the National Academy of Sciences of the United States of America*, *114*(20), 5300–5305. https://doi.org/10.1073/pnas.1700712114

Riebold, M., Mankuta, D., Lerer, E., Israel, S., Zhong, S., Nemanov, L., … Ebstein, R. P. (2011). All-trans retinoic acid upregulates reduced CD38 transcription in lymphoblastoid cell lines from autism spectrum disorder. *Molecular Medicine*, *17*(7–8), 799–806. https://doi.org/10.2119/molmed.2011.00080

Schneiderman, I., Kanat-Maymon, Y., Ebstein, R. P., & Feldman, R. (2014). Cumulative risk on the oxytocin receptor gene ( *OXTR* ) underpins empathic communication difficulties at the first stages of romantic love. *Social Cognitive and Affective Neuroscience*, *9*(10), 1524–1529. https://doi.org/10.1093/scan/nst142

Tang, Y., Epstein, M. P., Anderson, G. M., Zabetian, C. P., & Cubells, J. F. (2007). Genotypic and haplotypic associations of the DBH gene with plasma dopamine β-hydroxylase activity in African Americans. *European Journal of Human Genetics*, *15*(8), 878–883. https://doi.org/10.1038/sj.ejhg.5201838

Tost, H., Kolachana, B., Hakimi, S., Lemaitre, H., Verchinski, B. A., Mattay, V. S., … Meyer-Lindenberg, A. (2010). A common allele in the oxytocin receptor gene (OXTR) impacts prosocial temperament and human hypothalamic-limbic structure and function. *Proceedings of the National Academy of Sciences of the United States of America*, *107*(31), 13936–13941. https://doi.org/10.1073/pnas.1003296107

Tost, H., Kolachana, B., Verchinski, B. A., Bilek, E., Goldman, A. L., Mattay, V. S., … Meyer-Lindenberg, A. (2011). Neurogenetic effects of OXTR rs2254298 in the extended limbic system of healthy Caucasian adults. *Biological Psychiatry*, *70*(9), e37–e39. https://doi.org/10.1016/J.BIOPSYCH.2011.06.034

Vitoratou, S., Ntzoufras, I., Theleritis, C., Smyrnis, N., & Stefanis, N. C. (2015). Temperament and character dimensions assessed in general population, in individuals with psychoactive substance dependence and in young male conscripts. *European Psychiatry*, *30*(4), 474–479. https://doi.org/10.1016/j.eurpsy.2015.01.007

Wu, N., Li, Z., & Su, Y. (2012). The association between oxytocin receptor gene polymorphism (OXTR) and trait empathy. *Journal of Affective Disorders*, *138*(3), 468–472. https://doi.org/10.1016/j.jad.2012.01.009
